# Infant with diffuse large B-cell lymphoma identified postmortem with homozygous founder Slavic *RAG1* variant: a case report and literature review

**DOI:** 10.3389/fped.2024.1415020

**Published:** 2024-07-04

**Authors:** Tatiana P. Volodashchik, Ekaterina A. Polyakova, Taisia M. Mikhaleuskaya, Inga S. Sakovich, Aleksandra N. Kupchinskaya, Aliaxandr Ch. Dubrouski, Mikhail V. Belevtsev, Joseph F. Dasso, Dzmitry S. Varabyou, Luigi D. Notarangelo, Jolan E. Walter, Svetlana O. Sharapova

**Affiliations:** ^1^ Research Department, Belarusian Research Center for Pediatric Oncology, Hematology and Immunology, Minsk, Belarus; ^2^ Pathological Unit, N.N. Alexandrov National Cancer Centre of Belarus, Lesnoy, Belarus; ^3^ Division of Pediatric Allergy/Immunology, Johns Hopkins All Children’s Hospital, Saint Petersburg, FL, United States; ^4^ Division of Pediatric Allergy/Immunology, University of South Florida, Tampa, FL, United States; ^5^ Department of Geographical Ecology, Faculty of Geography and Geoinformatics, Belarusian State University, Minsk, Belarus; ^6^ Laboratory of Clinical Immunology and Microbiology, Division of Intramural Research, National Institute of Allergy and Infectious Diseases (NIH), Bethesda, MD, United States

**Keywords:** *RAG* deficiency, lymphoma, malignancy in SCID, infant, case report

## Abstract

**Background and aims:**

There is an increased risk of lymphomas in inborn errors of immunity (IEI); however, germline genetic testing is rarely used in oncological patients, even in those with early onset of cancer. Our study focuses on a child with a recombination-activating gene 1 (*RAG1*) deficiency who was identified through a screening program for Slavic founder genetic variants among patients who died with malignancy at an early age in Belarus.

**Results:**

We identified one homozygous founder *RAG1* variant out of 24 available DNA samples from 71 patients who developed lymphoma aged <3 years from the Belarusian cancer registry between 1986 and 2023. Our patient had an episode of pneumonia at 3 months of age and was hospitalized for respiratory distress, candida-positive lung disease, and lymphadenopathy at 14 months of age. The diagnosis of Epstein–Barr virus (EBV)-positive diffuse large B-cell lymphoma (DLBCL) was established. The patient had a normal lymphocyte count that decreased over time. One month after chemotherapy initiation, the patient died due to sepsis and multiple organ failure without a genetic diagnosis. In a retrospective analysis, T-cell receptor excision circles (TRECs) and kappa-deleting recombination excision circles (KRECs) were undetectable in peripheral blood.

**Conclusions:**

A targeted screening program designed to detect a Slavic founder variant in the *RAG1* gene among children revealed a 14-month-old Belarusian male infant with low TREC levels who died of EBV-driven DLBCL and complications of chemotherapy including infections. This case highlights how patients with IEI and recurrent infections may develop serious non-infectious complications, such as fatal malignancy. It also emphasizes the importance of early identification, such as newborn screening for severe combined immune deficiency. Earlier diagnosis of RAG deficiency could have prompted hematopoietic stem cell transplant well before the DLBCL occurrence. This likely would impact the onset and/or management strategies for the cancer.

## 1 Introduction

Patients with inborn errors of immunity (IEI) have a much higher risk of developing cancer than the general population, in the range of 4%–25% (1, 2). There is some variability regarding the type of malignancy and its association with specific IEI. In patients with IEI, malignancies may occur at any age, including childhood, but on average they tend to manifest earlier than in the general population. In addition, there is a narrower age range with higher incidence of hematological malignancies than in the general population (3). Increased susceptibility to malignant neoplasms in IEI patients is mediated by a combination of intrinsic and extrinsic factors. Genetic disorders associated with impaired cell differentiation or apoptosis, cytoskeleton function, lymphocyte co-signaling, metabolism, cytotoxicity, and disorders leading to increased genotoxicity, such as chromosome instability, defective telomere maintenance and DNA repair, along with extrinsic causes, such as transforming viral infections and chronic tissue inflammation, contribute to the malignancy development (4, 5).

The most common IEI associated with increased cancer predisposition include DNA repair defects and/or IEI with susceptibility to oncogenic herpes group virus infection. Ataxia telangiectasia, Nijmegen breakage syndrome, and Bloom syndrome are the most frequently encountered DNA repair defects. Beside malignancy predisposition, these patients also have distinctive syndromic features that may expedite the diagnosis. In patients with combined immunodeficiencies (CIDs) with Epstein–Barr virus (EBV) susceptibility, infection may be one of the main triggers for the development of malignancy. These disorders include autoimmune lymphoproliferative syndromes, X-linked lymphoproliferative syndrome type I and regulatory T-cell disorders, and malignancy, mostly lymphoma (6–10).

Lymphoma is the most common tumor type in patients with IEI, with the risk of lymphoid malignancies being overall 8–10-fold higher (11). Non-Hodgkin lymphomas [diffuse large B-cell lymphoma (DLBCL), marginal zone, and Burkitt lymphomas] account for the vast majority of lymphomas observed in patients with IEI (12), whereas leukemia, Hodgkin's lymphoma, and solid tumors are less common in this group of patients (13).

We previously described 18 Slavic patients who were homozygous for the deleterious *RAG1* p.K86VfsX33 (c.256_257delAA) variant. This study is the largest report in the literature of *RAG* variants within a geographically restricted population (14).

Here we report the case of a 14-month-old male infant affected by EBV-positive DLBCL in whom homozygosity for the same *RAG1* Slavic founder variant was identified postmortem after targeted Sanger sequencing of 24 available DNA samples out of 71 patients who developed lymphoma aged <3 years from the Belarusian cancer registry between 1986 and 2023. In addition, we performed a literature search of malignancy occurrence in *RAG* deficiency.

## 2 Results

### 2.1 Cohort analysis

We analyzed the data of the Belarusian cancer registry and found 71 patients (48 boys, 23 girls) who had been diagnosed with lymphoma before the age of 3 years in the period between 1986 and 2023. There were 48 non-Hodgkin's lymphomas, 9 Hodgkin's lymphomas, and 14 unspecified lymphomas (diagnosed before 1995). The age at clinical diagnosis was in the range of 1 month to 2 years 10 months (median 2 years 2 months). Of these patients, 38% were reported to be alive in 2024. Genetic material for DNA extraction was available in only 24 patients who comprised the study cohort. The cohort for sequencing included 17 boys and 7 girls, of whom 62.5% were alive at the time of the study (median age 11 years). The age at clinical manifestation of lymphoma in this group of patients ranged from 7 months to 2 years 10 months (median 2 years).

Homozygosity for the founder Slavic *RAG1* p.K86VfsX33 variant was found in 1 of 24 samples of available DNA.

Previous studies in the same cohort of 24 patients had identified one homozygous patient for the founder Slavic *UNC13D* variant [p.Arg782SerfsTer12] and two patients had disease-associated deleterious variants [p.Pro465ArgfsTer82 and p.His321Tyr] in the *FOXN1* gene. In total, 4 of the 24 patients were diagnosed with IEI.

### 2.2 Clinical case

A male infant was born full-term from non-consanguineous parents who originated from Brest (Western Belarus), after a second pregnancy and second delivery. The patient’s family history was unrevealing. He had received hepatitis B virus (HBV) (first dose) and bacille Calmette-Guérin (BCG) vaccine without adverse effects.

**Figure 1:**
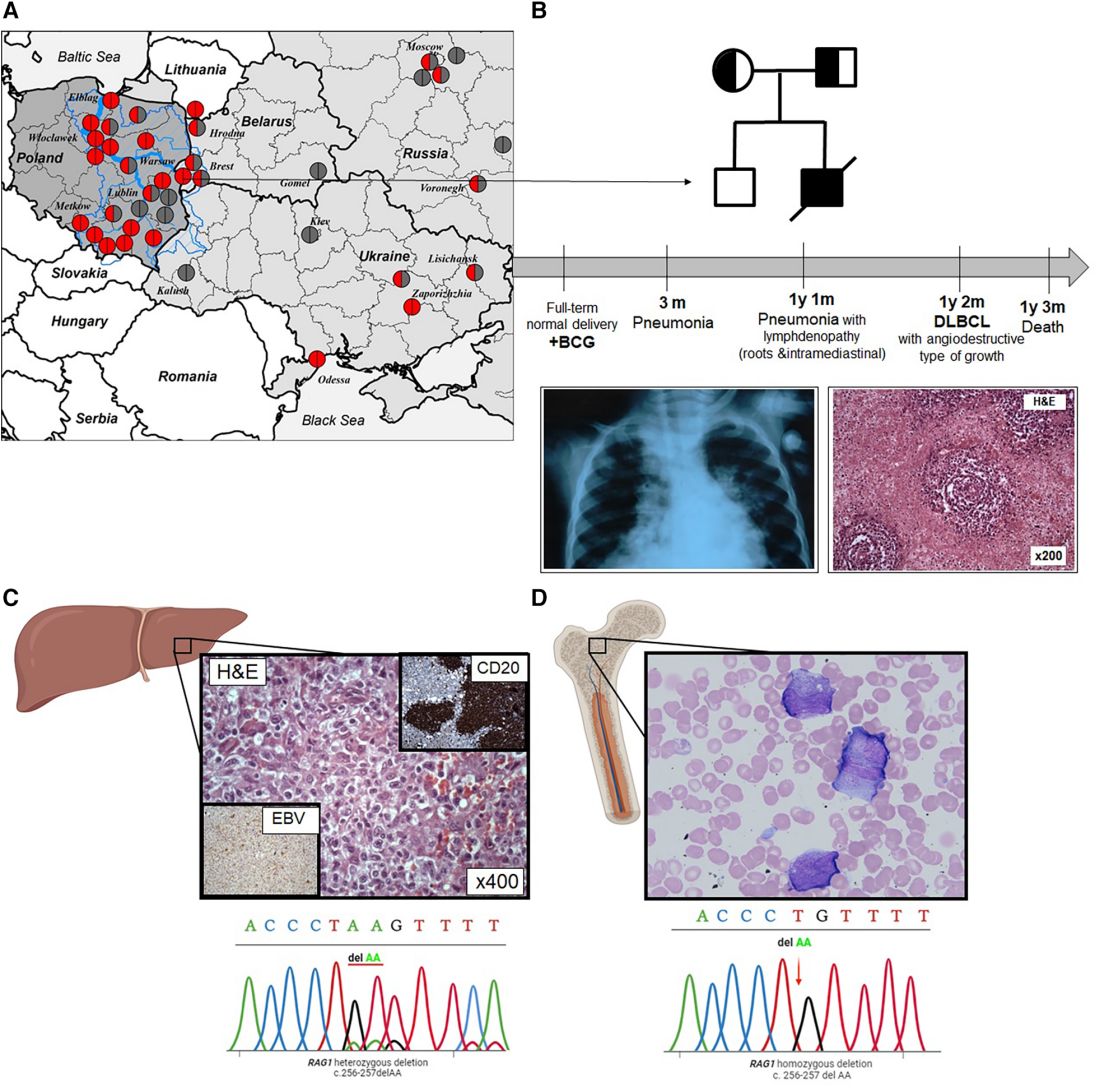
(**A**) Distribution map of Western and Eastern Slavic families with the *RAG1* p.K86VfsX33 variant in Slavic countries; the Vistula river basin is overlaid on the map of Poland. The blue line is the Vistula River basin and its largest tributaries (Bug, Narev, San, Wieprz, Pilica, and Muchowiec), the geographic area coincides with the region of the largest concentration of families where patients with p.K86VfsX33 homozygous variants were born. The birthplace of the patients was indicated by the location of the circles; homozygous p.K86VfsTer33 variant is represented by red circles; heterozygous p.K86VfsTer33 variant is half red/half gray; and other variants are gray. (**B**) Pedigree of the proband; the mother and father are carriers; deceased (line through). Timeline summarizing main turning point of patient's clinical history. Chest radiograph at the age of 3 months: pneumonia; liver biopsy at the age of 1 year 2 months: lymphoma with angiodestructive type of growth. (**C**) Liver biopsy: solid shut of lymphoma cells, some of them resemble Reed–Sternberg cells and electropherograms of Sanger sequencing of *RAG1* p.K86VfsX33 variant from lymphoma tissue. (**D**) Bone marrow smear: dissociation of neutrophil maturation cells and electropherograms of Sanger sequencing of *RAG1* p.K86VfsX33 variant in the bone marrow.

From the age of 3 months, the patient presented with a history of recurrent upper and lower (pneumonias) respiratory tract infections. At the age of 1 year 1 month, the patient was hospitalized because of fever, cough, dyspnea, lymphadenopathy, weakness, and refusal to eat. At physical examination, muscular hypotrophy of the second degree (weight 7.5 kg and height 75 cm), flabby skin, cyanosis of the nasolabial triangle, and respiratory distress were found. Chest radiograph showed bilateral polysegmental pneumonia (Figure 1B). A microbiological analysis of sputum found Candida.

An ultrasound examination showed hepato-(+3 cm)-spleno-(+3 cm)-megaly and lymphadenopathy. A lymph node biopsy was suggestive of DLBCL. Angiocentric lymphoid infiltrates of large, atypical cells and Hodgkin-like cells were accompanied by foci of coagulative necrosis. Large cells showed LCA, CD20, and patchy CD30 positivity. Immunostaining for EBV latent membrane protein was positive (Figure 1C). The lymphoma involved multiple lymph nodes (cervical, retroperitoneal, mediastinal, and abdominal) and multiple organs (liver, lungs, large and small intestines). Bone marrow investigation revealed dissociation of neutrophil maturation, hypochromia, and microanisocytosis of erythrocytes (Figure 1D).

Treatment was started according to the NHL-BFM-95 protocol with a dose reduction of one-third.

Laboratory investigation at the age of 1 year 1 month revealed progressive leukopenia (from 3,100 to 200 cells/µl), an imbalance in the ratio of stab and segmented neutrophils, and decreased hemoglobin (96 g/L). Lymphopenia (400 cells/µl), decreased T cells (300 cells/µl), and elevated activated T cells (41%) were detected. The percentage of B cells was elevated (33%), but the absolute number was slightly decreased (100 cells/µl). T-cell receptor excision circles (TRECs) and kappa-deleting recombination excision circles (KRECs) were undetectable (0 copies/10^6^ leukocytes for both) and DNA was isolated from archived patient peripheral blood samples obtained at the time of hospitalization (1 year 3 months).

During the course of the lymphoma treatment, the patient's condition worsened. He developed sepsis (*Pseudomonas aeruginosa* and *Enterococcus faecium*), fibrinous-purulent peritonitis, and intestinal perforation. Resection of the ileocecal angle was performed. During surgery, biopsies of the liver, ileum, cecum, and ascending colon were taken. Extensive necrosis in the center and multiple nodular proliferations of diffuse large B-cell lymphoma were found in the tissues of the liver and intestines.

The patient died at the age of 1 year 3 months due to septic shock and multiple organ failure, 1 month after starting chemotherapy.

At 18 years after his death, targeted Sanger sequencing revealed a homozygous deletion in *RAG1* gene (NM_000448.3) c.256_257del (p.Lys86ValfsTer33).

## 3 Discussion

The recombination-activating gene 1 (*RAG1*) and 2 (*RAG2*) encode lymphoid-specific proteins that are expressed during the early stages of T-cell and B-cell development and initiate the process of V(D)J recombination by introducing DNA double-strand breaks (DSBs). The process of V(D)J recombination generates diverse T-cell and B-cell receptors capable of recognizing millions of possible antigens (15). Genotype–phenotype correlation is strong, as null variants of *RAG1* and *RAG2* genes result in the T-B-severe combined immune deficiency (SCID) phenotype, whereas hypomorphic *RAG* variants have been associated with distinct clinical entities, including Omenn syndrome (OS) and combined immunodeficiency with granuloma and/or autoimmunity (CID/G-AI) with herpesvirus infections and lymphoproliferation (16).

However, in RAG1/RAG2-deficient patients, as well as in overall SCID patients, hematopoietic neoplasms are extremely rare. This is presumably explained by the high frequency of life-threatening infections that require hematopoietic stem cell transplantation (HSCT) early in life. Currently, there are only six published cases of malignancy in five patients with *RAG1* deficiency (Table 1) (17–21).

**Table 1:** Clinical data of patients with *RAG* deficiency and malignancies.

Patient No._gender_ethnicity	*RAG*				Age		Malignancy			Treatment	Follow-up	Reference
	Gene	Mutation	*RAG1* activity (% of WT), mean ± SE (16)	Phenotype	At onset	*RAG1* deficiency/malignancy	Type	Localization	Virus (blood/blocks)			
P1_F_Turkish_Germany	*RAG1*	p.R561H	2.0 ± 0.6	T−B− SCID	4 months	∼10 months/10 months	DLBCL	Lung, mastoid sinus	CMV tissue, EBV+tissue and blood	Anti-CD20 mAb, HSCT	Alive, 6 months at time of publication	(17)
		p.R561H	2.0 ± 0.6									
P2_F_Germany	*RAG1*	p.R314W	24.3 ± 5.2	T^low^B^low^ CID	1 year 5 months	3 years/2.5 years	Clonal T-cell lymphoproliferative lesion, DLBCL	Right tonsil	EBV+tissue and blood	Chemotherapy, anti-CD20 mAb, HSCT	Alive, 3 years at the time of publication	(18)
		p.R507W/p.R737H	15.9 ± 0.8/0.2 ± 0.0									
P3_M_Slavic_Romania/Austria	*RAG1*	p.K86VfsX33	2.7 ± 0.3	T−B− SCID	∼4 months	4 months/∼4 months	DLBCL	Liver	CMV blood, EBV+tissue and blood	Anti-CD20 mAb, modified CHOP, HSCT	Alive at the time of publication	(19)
		p.K86VfsX33	2.7 ± 0.3									
P4_M_China	*RAG1*	c.813T>A/c.870G>A	n.d.	T^low^B^low^ SCID	2 years	∼5 years/5 years	CLPD	Plantar skin	EBV− blood	Prednisone and methotrexate, HSCT	Alive, preparing for HSCT at the time of publication	(20)
		p.A740V	n.d.									
P5_F_Israel	*RAG1*	p.C335R	n.d.	T+B+CID	13 years	15 years/15 years	CLPD	Right cheek skin	EBV− tissue	Prednisone and methotrexate, local radiotherapy, HSCT	Died after HSCT	(21)
		p.C335R	n.d.									
P6_M_Slavic_Belarus	*RAG1*	p.K86VfsX33	2.7 ± 0.3	T^low^B^low^ CID	3 months	Postmortem/1 year 2 months	DLBCL	LN, liver, intestine	EBV+tissue and blood	Chemotherapy	Died on 2nd month of chemotherapy	Current case
		p.K86VfsX33	2.7 ± 0.3									

These patients had only two types of neoplasms: DLBCL (three cases) and cutaneous lymphoproliferative disease (CLPD) (three cases). CLPD manifested at a later age (2.5, 5, and 15 years) in patients with milder phenotype, which can be described as combined immunodeficiency or “leaky” SCID (20, 21). In contrast, DLBCL manifested earlier: in two cases, the diagnosis was established in the first year of life and occurred even in patients with typical SCID phenotype (17, 19). In only one patient with “leaky” SCID phenotype was DLBCL diagnosed at the age of 5 years (18). In the current case, DLBCL was established at the age of 1 year 2 months. Even though the patient had a history of severe infectious episodes and lymphopenia, a diagnosis of SCID was contemplated, but was not formally established during the patient's lifetime. EBV was detected in peripheral blood and tumor tissue in all four patients with DLBCL, including the current case. Rituximab therapy was initiated to rapidly control the EBV load. Patients with CLPD were treated with a combination of prednisone, methotrexate, and local radiation therapy (patient 5). All patients underwent HSCT except patient 4 who was being prepared for transplantation at the time of publication. Patient 5 did not recover owing to severe infection after HSCT.

Numerous lymphoma sequencing studies show that somatic mutations are often found in IEI genes. The most recurrently mutated genes in Hodgkin’s lymphoma and DLBCL include *TNFAIP3*, *SOCS1*, *ITPKB*, *B2M*, *KMT2D*, *ATM*, *TP53*, *ACTB,* and *IRF4* (22–24).

Many studies showed that RAG endonucleases are involved in the pathogenesis of lymphomas and leukemias. Dysregulation of RAG expression may cause chromosomal translocations, which are a hallmark of hematopoietic neoplasms (25). RAG endonucleases are able to bind to nonamer-like sequences (cryptic nonamer) and cleave at adjacent mismatches resulting from activation-induced cytidine deaminase (AID)-mediated deamination of methylated CpG sites (26), thereby generating genomic instability. Whole genome sequencing of cutaneous T-cell lymphomas (CTCLs) revealed that RAG binding sites flanked a significant number of deletion breakpoints (27). Since CTCLs occur in older adults, this suggests that dysregulated re-expression of the *RAG* genes may occur in mature CD4+ T cells, leading to tumorigenesis. Complex genomic rearrangements such as chromothripsis, in which DSBs are widespread, have also been found in CTCLs (27).

Somatic mutations in the *RAG1* and *RAG2* genes are rare in lymphogenesis and leukemogenesis. According to data from the catalogue of somatic mutations in cancer (COSMIC), out of 8,381 samples of hematopoietic and lymphoid tissue studied, only 24 (0.29%) had point mutations in the *RAG1* gene [https://cancer.sanger.ac.uk/cosmic, accessed May 2024]. These mutations have been found in acute myeloid leukemia, acute lymphoblastic B-cell leukemia, adult T-cell lymphoma/leukemia, plasma cell myeloma, diffuse large B-cell lymphoma, chronic lymphocytic leukemia-small lymphocytic lymphoma, mycosis fungoides-Sezary syndrome, and other cancers. A similar pattern is observed for the *RAG2* gene: *RAG2* somatic mutations have been reported in patients with Burkitt lymphoma, mycosis fungoides-Sezary syndrome, acute lymphoblastic B-cell leukemia, DLBCL, chronic lymphocytic leukemia, small lymphocytic lymphoma, and so on. Somatic point mutations in the *RAG2* gene were detected in 18 (0.21%) of 8,393 hematopoietic and lymphoid tissue samples studied.

Approximately 15% of human cancers worldwide are caused by oncoviruses. Human oncogenic viruses include EBV, HBV, hepatitis C virus (HCV), high-risk human papillomaviruses (HPVs), human T-cell lymphotropic virus-1 (HTLV-1), Kaposi sarcoma-associated herpesvirus [KSHV; also known as human herpesvirus 8 (HHV-8), and Merkel cell polyomavirus (MCPyV)] (28). However, oncovirus infection alone is not sufficient to cause cancer. Within the context of multistep carcinogenesis, viral infection provides only a subset of the required oncogenic hits (29).

To successfully evade the immune response, oncoviruses have evolved powerful anti-apoptotic and proliferative programs. There are several basic oncogenic mechanisms. First, viruses encode proteins that are able to subvert, in a dominant manner, host-signaling mechanisms that regulate cell growth and survival. Second, recognition of viral genomes or replicative intermediates by the host leads to induction of the DNA damage response (DDR), which many oncoviruses need for their replication. Third, chronic inflammatory responses to persistent viral infection cause the formation of reactive oxygen species (ROS) that promote the acquisition of mutations (29).

Sheng et al. demonstrate that in a group of 329 patients with DLBCL, only 2.4% were EBV positive (23). According to our data, all patients with RAG1 deficiency had EBV-associated DLBCL. Evidence of the association between EBV positivity and mutation burden is mixed. Some studies have shown a strikingly lower number of somatic mutations in EBV-positive Hodgkin’s lymphomas (30).

A targeted screening program for searching the Slavic founder variant in *RAG1* gene among Belarusian children who developed lymphoma aged <3 years revealed a 14-month-old Belarusian boy with low TREC levels who died of EBV-driven DLBCL and complications of chemotherapy including infections. This case highlights how patients with IEI and recurrent infections may develop serious non-infectious complications, such as fatal malignancy. It also stresses the importance of early identification, such as newborn screening for SCID, especially in the regions with domination of founder mutations. In addition, our case underlines the importance of resequencing the genomic DNA in patients with malignancy and somatic mutations in IEI genes.

## Data Availability

The original contributions presented in the study are included in the article/Supplementary Material, further inquiries can be directed to the corresponding author.
